# The value of chest X-ray and CT severity scoring systems in the diagnosis of COVID-19: A review

**DOI:** 10.3389/fmed.2022.1076184

**Published:** 2023-01-12

**Authors:** Naif Ali A. Majrashi

**Affiliations:** Diagnostic Radiography Technology (DRT) Department, Faculty of Applied Medical Sciences, Jazan University, Jazan, Saudi Arabia

**Keywords:** review, COVID-19, severity score index, X-rays, CT scans (CT)

## Abstract

Coronavirus disease 2019 (COVID-19) is caused by a coronavirus family member known as severe acute respiratory syndrome coronavirus-2 (SARS-CoV-2). The main laboratory test to confirm the quick diagnosis of COVID-19 infection is reverse transcription-polymerase chain reaction (RT-PCR) based on nasal or throat swab sampling. A small percentage of false-negative RT-PCR results have been reported. The RT-PCR test has a sensitivity of 50–72%, which could be attributed to a low viral load in test specimens or laboratory errors. In contrast, chest CT has shown 56–98% of sensitivity in diagnosing COVID-19 at initial presentation and has been suggested to be useful in correcting false negatives from RT-PCR. Chest X-rays and CT scans have been proposed to predict COVID-19 disease severity by displaying the score of lung involvement and thus providing information about the diagnosis and prognosis of COVID-19 infection. As a result, the current study provides a comprehensive overview of the utility of the severity score index using X-rays and CT scans in diagnosing patients with COVID-19 when compared to RT-PCR.

## 1. Introduction

Coronavirus disease 2019 (COVID-19), which has become a global pandemic in early 2020 (1), is caused by a member of the coronavirus family known as a novel SARS-CoV-2 (2–4). The most common clinical presentation of SARS-CoV-2 involves fever, cough, dyspnea, and respiratory tract symptoms. There have been over 6 billion worldwide tests for COVID-19. SARS-CoV-2 testing is essential not only for the diagnostics and treatment of COVID-19 infection by medical institutions but also as a prerequisite for major semi-normal economic and social activities such as international flights and social events. RT-PCR based on nasal or throat swab sampling, bronchoalveolar lavage, or tracheal aspirate (5) is considered the main laboratory test to confirm the quick diagnosis of COVID-19 infection. However, a small but significant proportion of false-negative RT-PCR results has been reported (6). There is evidence reporting that the sensitivity of the RT-PCR test is approximately 50–72% (7). It has been suggested that this percentage of sensitivity may be attributed to the low viral load present in test specimens or laboratory errors (8, 9). In contrast, for example, chest CT (CCT) has demonstrated approximately 56–98% of sensitivity in diagnosing COVID-19 at initial presentation and has been suggested to be helpful in rectifying false negatives obtained from RT-PCR (10). Chest X-rays (CXR) and CT scans have been suggested to predict COVID-19 disease severity by showing the score of lung involvement and, therefore, providing an idea about the diagnosis and prognosis of COVID-19 infection (11, 12). Therefore, the current literature review paper aims to provide an overview of the clinical value of the severity score index using X-rays and CT in diagnosing patients with COVID-19 compared to RT-PCR. A secondary aim is to determine if the imaging severity score findings can correlate with demographics and other variables.

Previous review studies focused on laboratory approaches (13), commercial technologies (14), or detection advancements (15) for detecting SARS-CoV-2 and have extensively discussed the role of RT-PCR and diagnostic imaging in general including, X-rays, CT, and ultrasound in testing COVID-19 infection (16) but not the severity score index. While RT-PCR is the gold standard for virus detection, it has been shown to have diagnostic errors, and there is a clear demand for faster, less expensive point-of-care tests. The possible approach of using the severity score index on CXR and CT scans that could be created to meet this demand is outlined in this review. This overview may be useful to a wider audience concerned with the understanding and enhancement of COVID-19 testing that will one day help put an end to the epidemic. This overview will further enable physicians to better understand the added value of the severity score index using X-rays and CT in diagnosing patients with COVID-19. An extensive review of the literature published in English was carried out using the PubMed and Google Scholar databases. The search terms included COVID-19 (OR SARS-CoV-2, OR CORONA VIRUS), chest, X-rays, computed tomography (OR CT), and severity scores (OR severity index, OR severity scoring system).

## 2. Related work

### 2.1. Overview of viruses

Viruses are defined as nanometer-scale infectious pathogens that can only live and propagate inside the cells of a living host (13). Viruses can infect a wide variety of organisms, including humans. It has been suggested that viruses are mobile genetic components of cellular origin that have coevolved with their hosts (17). A complete virus particle is called a virion, which is responsible for transmitting its DNA or RNA genome into the host cell so that it can be transcribed and translated. The viral genome, along with related basic proteins, is wrapped in a symmetric protein capsid. The nucleic acid-associated protein nucleoprotein and the genome create the nucleocapsid. Viruses are categorized based on the nucleic acid used for encoding genetic material, including DNA or RNA (13, 14). Double-stranded DNA is typically found in DNA viruses, but not single-stranded DNA. Although most viruses can only infect members of their own species, others such as influenza A and C can spread between species. For example, coronaviruses are known to change and spread from one species to another. Coronaviruses, such as Middle East Respiratory Syndrome Coronavirus (MERS-CoV), which was first reported in 2012 in Saudi Arabia (transmitted from dromedary camels) and caused 34.4% mortality in the Middle East, and SARS-CoV-2, which first appeared in China in 2022, are recurring in humans. Coronaviruses are enclosed positive sense single-strand RNA viruses. SARS-CoV-2 is thought to be related to bat and pangolin coronaviruses (18). Coronaviruses create global pandemics due to their high transmissibility and mortality, such as the SARS-CoV-2 outbreaks in 2002–2003 and 2019 (19).

### 2.2. Overview of COVID-19 and its effects

COVID-19, a disease that emerged from Wuhan, Hubei Province (China), has infected millions of people and become a global pandemic on 11 March 2020 (20). Since then, the world has been coping with its ongoing outbreak (2). It has been spreading globally, with up to 650,140,594 active infections and a total death toll of 6,647,157.^1^,^2^ The coronaviruses, which are given this name for their crown-like appearance due to surface spikes, are divided into four broad classes (alpha, beta, gamma, and delta). Due to the enormous development of COVID-19 cases, many advanced countries’ health systems are collapsing. They lack ventilators and testing kits. Many countries have asked their people to stay indoors and avoid gatherings. COVID-19 is defined as an illness caused by a member of the coronavirus family, SARS-CoV-2, that shares up to 70% of amino acid identity, sequence identity, and structural similarity (2–4). SARS-CoV-2 has had a severe influence on the world economy, causing stock market instability. Due to lockdowns, the coronavirus epidemic has caused job losses and increased global unemployment. Government limitations will cost the travel and hospitality industries $100 billion between 2020 and 2021 (13). SARS-CoV-2 restrictions are a vital step, but they are not the only way to manage the virus. There is a wide spectrum of clinical manifestations, from asymptomatic to fatal. The clinical presentation of SARS-CoV-2 involves fever and respiratory tract symptoms. Most patients with COVID-19 recover from these symptoms quickly. The respiratory tract system is the most relevant clinical feature in patients with COVID-19. Other body systems have also been affected by the COVID-19 infection. For example, a recent study (21) has found that nearly 37% of patients with COVID-19 presented with central nervous system (CNS) symptoms and 9% with peripheral nervous symptoms. SARS-CoV-2 has also been shown to invade human cells, based on laboratory methods, and has the potential to attack the CNS (22). SARS-CoV-2 protein has been found in brain vascular endothelium. A previous study (23) demonstrated that SARS-CoV-2 can be detected in the cerebral-spinal fluid (CSF) in patients with COVID-19. It has been suggested that SARS-CoV-2 enters the brain from the cribriform plate along the olfactory tract or trigeminal pathway (24), penetrates the olfactory mucosa, leading to a loss of smell, passes the blood-brain barrier (BBB) due to BBB instability caused by inflammatory cytokines *via* monocytes (25), and reaches brain tissue *via* midline structures around the third and fourth ventricles and circumventricular organs (CVOs) (26). A previous study (27) showed that nearly 37% of hospitalized patients with COVID-19 presented neurological symptoms such as dizziness, headache, infarction, and impaired consciousness. Previous studies also demonstrated microstructural changes in patients with cerebral viral infections, including human immunodeficiency virus (HIV) and herpes simplex virus (HSV) non-invasively (28, 29). In addition, previous studies (30–32) reported that patients with COVID-19 presented with various neurological diseases, including encephalitis, stroke, micro-hemorrhage, hemorrhage posterior reversible encephalopathy, and cerebral venous embolism, in 12 European hospitals. Furthermore, previous studies (33, 34) discussed that COVID-19 can cause demyelination, neurodegeneration, and cellular senescence, leading to accelerating brain aging and exacerbating neurodegenerative pathology. Because the respiratory system is mostly affected by COVID-19, two imaging techniques, including CXRs and computed tomography (CT), are strongly recommended for COVID-19 pulmonary infections in the initial evaluation (35).

There is also evidence (24, 36) demonstrating that patients with COVID-19 present with anosmia, cognitive and attention deficits, seizures, depression, psychosis, anxiety, and even suicidal behavior and that these symptoms present before, during, and after respiratory symptoms. A previous study (37) also found a link between COVID-19 and mood (depressive and anxiety symptoms) changes, as well as that the social and work disruptions caused by the pandemic COVID-19 were associated with mental health impairments. Holmes et al. (37) have found that UK citizens are more concerned about how societal changes will impact their psychological and financial wellbeing, than becoming ill with the virus (37). Furthermore, previous studies (38–40) found a relationship between loneliness and the observed mental health effects of the COVID-19 pandemic. Moreover, previous studies (41, 42) investigated mood responses in adolescents during the period of COVID-19 restrictions and found that scores for tension, depression, anger, fatigue, and confusion were elevated during the periods of the pandemic restrictions. They also found that women reported more negative mood scores than men as did participants aged less than 25 years compared to those above 56 years, suggesting that mood disturbance during the period of COVID-19 restrictions may be associated with an increased risk of psychopathology. A systematic review study (43) has also demonstrated that the general population in the USA, Europe, and the Middle East reported high rates of symptoms of depression, anxiety, psychological distress, post-traumatic stress disorder, and stress, ranging from 6.33 to 81.9%, with women showing the greatest effects. Together, these previous studies indicated a relationship between the pandemic and mood symptoms in patients with COVID-19 and that the necessary public health arrangements surrounding the pandemic have serious implications for community mental health.

### 2.3. Diagnosis (detection methods) of COVID-19

There have been over 6 billion tests for COVID-19 worldwide (13). SARS-CoV-2 testing is essential not only for the diagnostics and treatment of COVID-19 infection but also as a prerequisite for major semi-normal economic and social activities. The current diagnostic tests available for dealing with COVID-19 encompass a trio of complementary approaches including nucleic acid amplification, serology, and tomography, ranging from quick clinical judgments to screening vast populations (15). Molecular detection of the virus’s nucleic acids (RT-PCR) has been suggested as a sensitive and specific molecular test to identify the existence of viral nucleic acids. The ORF1b (RdRp), N, E, and S genes are popular targets for commercially available viral nucleic acid detection kits (18, 44). Depending on the method used, detection can take anywhere from a few minutes to many hours (45, 46). Numerous variables can impact molecular detection. Thus, several detection methods should be utilized to confirm a COVID-19 diagnosis. Molecular diagnosis may be supplemented by SARS-CoV-2 serological assays identifying antibodies to the N or S proteins, especially in the late stages after disease onset or for retrospective studies (47, 48). In contrast, in recent research, approximately 57% of individuals with SARS-CoV-2 infection had abnormal lung imaging (49). According to these findings, imaging diagnostics may serve as a supplementary technique in helping to diagnose the severity of COVID-19. One of the quickest methods to diagnose patients with COVID-19 is by looking for signs of the disease in radiographs and other imaging techniques. In terms of imaging, the most commonly used diagnostic methods are CXR and CT scans. It has been reported that hospitals in Italy and the United Kingdom are adopting CXR as a primary triage technique due to long RT-PCR turnaround times (50).

Chest X-ray, a low-cost and easy-to-use imaging technique, is also used to screen for and diagnose acute respiratory tract infections of the respiratory system. In patients with COVID-19, there is evidence showing that CXR is very helpful in detecting intermediate to advanced stages of COVID-19 but is of limited value in detecting the early stages of COVID-19 (51–53). CXR has also been suggested to be a useful diagnostic tool for monitoring the rapid progression of lung involvement in COVID-19, particularly in patients admitted to intensive care units (ICUs) (54). In addition, a previous study (55) stated that despite the CXRs’ evident abnormalities of COVID-19 pulmonary infection, X-ray images were normal in more than half of the patients. Furthermore, a previous study (56) found that the positive imaging features, including ground glass opacities, are only evident in CCT scans but not in CXR images. A previous study (57) discussed that multi-lobar involvement and rounded and peripheral airspace opacities were described in the largest case series of chest imaging, which included 21 patients (58). As stated by a previous study (57), opacities are most often characterized as having ground glass attenuation (57%) and mixed attenuation (29%), suggesting that findings may be missed on CXRs, but not on CCT scans due to the prevalence of ground-glass opacities. CCT scan has been shown to play an indispensable role in predicting COVID-19 pneumonia. CCT scan has also been suggested to give important and valuable information on the diagnosis, follow-up, management, and prognosis of patients with COVID-19 (59), especially in developing countries (12). CT findings have also been shown to be present early, even before the onset of COVID-19 symptoms (11), suggesting that CT is considered the main investigator for COVID-19 infection. CCT, particularly non-contrast CCT, has been used in patients with a confirmed COVID-19 infection (60). Since the pandemic, the clinical indications for using CCT (non-contrast) have been continuously evolving. A previous study (61) stated that CT was widely used as a supporting tool in the diagnosis of COVID-19 in China, during the early phase of the outbreak. However, later, guidelines from China’s National Health Commission do not include CCT findings or any imaging findings in the diagnostic criteria (62). The American College of Radiology (ACR) also did not recommend using CCT as a first-line imaging technique to screen for COVID-19 infection. However, Ng et al. (55) have discussed that in persons under investigation (PUI) for COVID-19, the number of CT scans performed may increase. In addition, there is evidence (5, 63) describing and distinguishing COVID-19 from other viral pneumonia at CCT with high accuracy and sensitivity but low-to-moderate specificity, by looking at the changes in the imaging features over time. Viral types of pneumonia have been suggested to have a wide variety of CT imaging features and presentations (55). However, in COVID-19 pneumonia, some of these features are uncommon or rare. Such examples of these features include bronchial wall thickening, bronchial mucus plugs, tree-in-bud opacities, and other small nodules. Therefore, viral types of pneumonia have a range of imaging criteria, not all of which are typical for COVID-19. Furthermore, the WHO advised using chest imaging as part of the diagnostic workup of COVID-19 disease in the following cases: when there is a clinical suspicion of COVID-19 with initial negative RT-PCR testing or when the RT-PCR test is not available or its results are delayed (64). Although imaging data may be inconclusive in determining the cause of an illness, radiologists should be on the lookout for nodular and peripheral ground-glass opacities in the setting of travel history or exposure (57).

## 3. Comparative analysis

### 3.1. RT-PCR vs. imaging (X-rays and CT scans) in the diagnosis of COVID-19

At the present, RT-PCR based on nasal or throat swab sampling, bronchoalveolar lavage, or tracheal aspirate (5) is considered the main laboratory test to confirm the quick diagnosis of COVID-19 infection; radiographic imaging, particularly CT, may be considered a valid tool in the initial diagnosis of COVID-19 pulmonary infection. Although RT-PCR for COVID-19 infection is considered a powerful tool, a small but significant proportion of false-negative results have been reported (6). A previous study described that a 34-year-old male was found to be negative for COVID-19 after undergoing four consecutive RT-PCR tests of his pharyngeal swab. On admission, CCT showed patchy ground-glass opacity, which rapidly advanced to segmental mixed consolidation and ground-glass opacity 3 days later and cleared in the left upper lobe but displayed multifocal ground-glass opacities 7 days later and resolved within 2 weeks. Five days following admission, a positive RT-PCR result was found, suggesting the value of CCT to detect early alteration of COVID-19 in cases where RT-PCR testing provides negative results, despite the fact that it is challenging to differentiate COVID-19 pneumonia from other viral pneumonia on CT features alone (65). It has been reported that the overall RT-PCR positive rate for throat swab samples is between 30 and 60% (66). There is evidence reporting that the sensitivity of the RT-PCR test is approximately 50–72% (7), possibly attributable to the low viral load present in test specimens or laboratory error (8, 9). It has been suggested that these false negatives have the potential to overload the current supply of testing kits and hinder quarantine efforts and repeat testing (10). SARS-CoV-2 has been found in throat swabs, posterior oropharyngeal saliva, nasopharyngeal swabs, sputum, and bronchial fluid; however, the viral load is larger in lower respiratory samples (44, 67, 68). Even when respiratory tests were negative, intestinal or blood samples had viral nucleic acid (47). Finally, before disease onset, viral load may decrease (68). It has been reported that the total positive rate of RT-PCR for throat swab samples ranges from 30 to 60%, resulting in undiagnosed patients who could potentially infect a large number of otherwise healthy individuals. Together with the low sensitivity of RT-PCR, the relatively long turnaround time (TAT) for viral testing implies that a large number of patients infected with SARS-CoV-2 would not be quickly identified and may not be appropriately managed (69). It has been suggested that the accuracy of RT-PCR results is affected by several factors, including the sample source, respiratory tract viral load, timing of sample acquisition, the intrinsic features, and quality of the testing kits, and the procedure (46). It has been suggested that even a small increase in any of COVID-19’s’ crucial parameters, such as accuracy, sensitivity, specificity, time to results, and cost per test, could have a profound effect on people’s daily lives in a wide variety of countries. Therefore, other detecting methods were applied. In contrast, CCT non-contrast, which has demonstrated approximately 56–98% of sensitivity in diagnosing COVID-19 at initial presentation, has been suggested to be helpful in rectifying false negatives obtained from RT-PCR (10). In more detail, CCT can reveal areas of bilateral peripheral ground-glass opacity and consolidation in multiple lobes progressing to “crazy-paving” patterns (55, 70). Short-term CT follow-up of a patient with COVID-19 has been reported. A previous study described the temporal evolution of 21 patients who recovered from COVID-19 (71). Out of 24 CT scans performed early (0–4 days following symptom onset), 17% had no lung opacities, 42% had focal ground-glass opacity or consolidation, and 42% had multifocal lung opacities. Pulmonary opacities were most common in the periphery in around half of the cases. Lung opacities worsened over the course of a disease’s middle stages (days 5–13 on a CT scan). The peak stage of lung involvement was marked by the appearance of a crazy-paving pattern in the lungs (19%), the development of new or increased consolidation in the lungs (91%), and an increased incidence of bilateral and multi-lobar involvement (86%). CT scans performed at a later stage (14 days or more) showed variable degrees of clearance but no resolution for at least another 26 days. Using the RT-PCR test as an independent and sole tool for diagnosing individuals with suspected COVID-19 pulmonary infection would be debatable, so complementary tools, such as X-rays and CT scans, have been proposed to participate in the screening and diagnosis of COVID-19 infection in addition to the PCR test and can significantly improve the accuracy of diagnosis in pulmonary viral diseases (7, 12). Although imaging manifestations resemble those seen in viral cases of pneumonias, such as peripheral multifocal ground-glass opacities and consolidation, and lack specificity for a COVID-19 diagnosis on imaging grounds, it has been suggested to use X-rays and CT severity scores to provide objective assessment about the extension of the lung opacities, which could be used as an imaging surrogate for disease burden (54, 69, 72). The severity score or index is defined as a scoring system used to assess lung involvement and changes caused by COVID-19 pulmonary infection (12). This approach is suggested to expedite the identification and management of patients with severe disease in specific instances.

### 3.2. The role of the severity scores of CXRs and CT scans (CXR-SS and CCT-SS) in the diagnosis of COVID-19

COVID-19 has infected around 1.8 million people and caused the deaths of approximately 114,698 worldwide. Most countries lack sufficient diagnostic tools due to the rising number of reported cases every day. Therefore, it is critical to accurately stratify patients with COVID-19 according to the severity of their diseases in order to allocate resources effectively (73). Specifically, the value of peripheral oxygen saturation (SpO2) is one of the first measures checked for each patient on admission; it often reflects the degree of lung function impairment. The requirement to transfer a patient with COVID-19 to an ICU is mostly determined by their SpO2, in addition to concurrent comorbidities (74). This, however, has been suggested to be achieved by imaging. CXR has been proposed to predict COVID-19 disease severity by showing the score of lung involvement and therefore providing an idea about the prognosis of COVID-19 infection. A recent previous study (75) has introduced the CXR scoring system for quantifying the severity and progression of lung abnormalities in COVID-19 pneumonia. Another previous study (76) assessed the extent of COVID-19 pulmonary abnormalities on CXR in large data set (926 consecutive patients) using a 0–3 semiquantitative severity score in 1-point increment on 6 lung zones (range 0–18), correlated it with clinical data, and tested its interobserver agreement. This study showed that interobserver agreement was found to be moderate to almost perfect, and associations with clinical parameters were significant (negative correlation with blood oxygen saturation but positive correlation with white cell count, lactate dehydrogenase, and C-reactive protein), suggesting that CXR might be further integrated into the classification of patients with COVID-19. This CXR scoring system, which is known as the *Brixia* score, was introduced in late March 2020 due to the high number of confirmed COVID-19 cases and a high mortality rate of 9.3% in Italy. The scoring system is a very useful tool for ranking the stratification of COVID-19 infection, suggesting confirmation of the use of the CXR scoring system in many countries (54). The CXR scoring system also provides information on pulmonary involvement based on an 18-point severity scale according to the extent and characteristics of lung abnormalities. Even though CXR is considered not sensitive for the detection of pulmonary involvement in the early stage of the disease, a previous study (54) suggested that CXR can be a useful diagnostic tool for detecting the rapid progression of lung abnormalities in infected patients with COVID-19, particularly in intensive care units (ICUs). The CXR scoring system is a simple five-point grading tool that was proposed in 2015 and was designed for non-radiologist clinicians. The goal of this scoring system was to facilitate the clinical grading of CXR reports into five different severity categories in hospitalized patients with acute respiratory infections.

### 3.3. CCT severity score index

The severity of COVID-19 can be ascertained from previous imaging findings, which supports the clinician’s clinical judgment and ensures effective and timely management (70). The severity of COVID-19 can also affect the prognosis of the disease in critically ill patients, allowing appropriate selection of early involvement in intensive care. The CCT has also been suggested to predict COVID-19 disease severity by showing the score of lung involvement. Emanuel et al. (72) proposed the use of the CCT severity score to categorize the severity of CT findings in the chest. The score is based on a technique developed in 2005 to monitor the spread of the SARS virus. For this scale, lung opacification serves as a proxy for the degree to which the lungs are affected. Each lung has 18 segments, and these segments are further subdivided into 20 regions using the CCT severity score. For example, the anteromedial basal segment of the left lower lobe is further divided into basal and anterior segmental regions, and the posterior apical segment of the left upper lobe is further subdivided into posterior and apical segmental regions. Parenchymal opacification scores could range from 0 (no engagement) to 2 (extreme involvement), with 0 indicating no involvement and 2 indicating 100% involvement in each location. The final score, from 0 to 40, is calculated by adding up the points for each of the 20 different areas of the lungs. Several previous studies (77, 78) have explained how pulmonary involvement in the CCT images was assessed using two methods, visual and software using deep learning algorithms for quantitative assessments. Currently, CCT is considered the most effective method for lung abnormality detection in the early stage of the disease and quantitative assessment of the severity of COVID-19 pneumonia (70, 79). CT severity score or index is defined as a scoring system used to assess lung involvement and changes caused by COVID-19 pulmonary infection (12). The scoring system is based on an approximate estimation of pulmonary involved areas. The right lung has three lobes, while the left lung has two lobes. Thus, each of the five lung lobes has been given a score from 1 to 5 based on a visual scoring (12), where number 1 represents less than 5% lobar involvement, number 2 represents 5–25% lobar involvement, number 3 represents 26–50% lobar involvement, number 4 represents 51–75% lobar involvement, and finally, number 5 represents more than 75% lobar involvement. All individual lobar scores are summed together to give a final score with a total score of 25 (69). According to a previous study (64), a score of 7 or less (out of 25) is mild severity, while from 8 to 17 is moderate, and above 18 is considered severe. There is evidence showing that a higher CT severity score was significantly correlated with male gender, older age groups, and a likely positive PCR test (12). A recent prospective study (80) has also found that the severity of COVID-19 was associated with higher age, male sex, and higher BMI. The CCT severity score index of lung involvement in the acute phase was also associated with restriction and a reduction in diffusion capacity in follow-up. In contrast, a recent study has found no differences in the severity score between sexes in the Mexican-Mestizo population grouped by age (81). Altogether, these previous studies support the correlation between the imaging (CXR and CT) severity score index and demographics.

Modern computational methods, such as deep learning or machine learning, are used for disease identification and play a crucial role in the screening of emergency cases. Using machine learning (or artificial intelligence (AI)-driven tool) has been suggested to have a number of advantages in the diagnosis of COVID-19 infection (82–86). It can assess several cases at the same time, be rapid, and have greater availability, making it highly useful in hospitals with no or limited testing kits and resources (87). Furthermore, since CXR is so important in today’s healthcare system, radiology imaging systems are available in every hospital, making radiography-based approaches more convenient and accessible. Nowadays, deep learning techniques are being used by researchers to recognize certain traits in CXR and CT images of patients with COVID-19. A previous study (87) developed a deep learning model (CoroNet model) to assist radiologists and doctors to detect patients with COVID-19 using CXRs. This study showed that after training and testing on the prepared dataset, the CoroNet deep learning model achieved an overall accuracy of 89.6%, with a 93% precision rate and a 98.2% recall rate for COVID-19 cases for four cases: COVID vs. pneumonia bacterial vs. pneumonia viral vs. normal. This approach achieved a 95% accuracy in classifying data into three classes (COVID, pneumonia, and normal), suggesting that initial findings from this study show promise. Another recent previous study (88) classified COVID-19 as normal, pneumonia-bacterial, and pneumonia-viral with 83.5% accuracy. In addition, a previous study (89) has reported a deep learning architecture for COVID-19 detection from CXR images and built a dataset of 5,000 COVID-19 CXRs with a radiologist’s help (using images from two datasets). This study found that most machine learning models achieved a specificity rate of around 90% and a sensitivity rate of around 98% on the COVID-X-ray-5000 dataset. Furthermore, a recent systematic review discussed AI-guided methods for the detection and screening of COVID-19 infections using cough sounds (86, 90). This previous review focused on AI-guided tools that use machine learning algorithms to evaluate cough sounds for screening for COVID-19. In CT scans, a previous study (91) designed a convolutional neural network (CNN)-tailored deep neural network (DNN) that can train or test CT scans. The study achieved 96.2% accuracy (AUC = 0.9808, false negative rate = 0.0208). Other than COVID-19, deep learning has been implicated in several chest diseases. For example, a previous review study (92) has discussed machine learning techniques for analyzing CXR images for screening cardiopulmonary abnormalities, particularly tuberculosis (TB). In addition, Shiraishi et al. (92) have developed a digital image database of 247 chest radiographs with and without a lung nodule in general. They investigated the characteristics of image databases to see if they could be used in various digital image research projects. A receiver operating characteristic (ROC) analysis was used to assess radiologists’ recognition of single lung nodules in the database. The ROC analysis demonstrated that the database contains a wide variety of nodules, with the area under the curve (Az) values ranging from 0.574 to 0.991 across the five categories of instances. Altogether, these studies support the role of AI including machine learning and deep learning, in the diagnosis of pulmonary diseases including, COVID-19.

## 4. Discussion

There is a dearth of literature reviews on the value of the severity score index, a newly recognized imaging approach, for diagnosing COVID-19. This study is the first to review the role of the severity scoring system in the diagnosis of COVID-19 infection. In the current review, Supplementary Table 1 summarizes most of the recent previous studies demonstrating the approach of the CXR and CT severity score index in diagnosing COVID-19. Almost all studies (except three studies) employed a cross-sectional design, and the primary analyses were significant. Most of the enrolled studies used a CT scan to calculate the severity score index based on the percentage of lung involvement in each patient with the help of expert radiologists. In most of these CT studies, each of the five lung lobes was assessed for the degree of involvement and classified as none (0%), minimal (1–25%), mild (26–50%), moderate (51–75%), or severe (76–100%). Notably, five studies showed that the most common CT features in COVID-19 cases were ground glass patches, followed by subpleural linear abnormality and air bronchogram (consolidation). Other radiographic features such as pleural effusion were also seen, but they were less common. The results have reported that there were a large number of patients who had 6-10 total severity scores out of 20, suggesting that most of the patients with COVID-19 in these studies were mildly affected by SARS-CoV-2. Importantly, most of the studies have demonstrated that CXR and CT severity scores were significantly correlated with demographics and laboratory results including hematological and inflammatory biomarkers. The severity scores were higher in men, and men aged 50 years or older and women aged 80 years or older with coronavirus disease in 2019 showed the highest CXR or CT severity scores or highest risk of developing severe lung disease. Higher severity scores were associated with positive PCR tests, lymphopenia, and increased serum CRP, d-dimer, and ferritin levels. This review may suggest that based on the previous studies, the severity score index may be considered an alternative way of diagnosing COVID-19 or its severity instead of RT-PCR, especially in countries where laboratory kits are not available. However, the results from these previous studies are based on different datasets, and therefore, they cannot be taken for a straight comparison.

## Author contributions

NM contributed to the writing and review of the literature and read and approved the final manuscript.

## Footnotes

1
https://www.worldometers.info/coronavirus/

2
https://covid19.who.int/

## Supplementary material

Supplementary Table 1 Characteristics of enrolled most common and recent studies demonstrating chest severity score (CSS) in COVID-19.
