# Supplementary material for: The value of chest X-ray and CT severity scoring systems in the diagnosis of COVID-19: A review
Source: Front Med (Lausanne). 2023 Jan 12;9:1076184. doi: 10.3389/fmed.2022.1076184 (PMC9877460; doi:10.3389/fmed.2022.1076184)
Supplement: Supplementary Table 1 — Characteristics of enrolled most common and recent studies demonstrating chest severity score (CSS) in COVID-19. [file Table_1.docx]

**Table 1**: Characteristics of enrolled most common and recent studies demonstrating chest severity score (CSS) in COVID-19

| **Authors** | **Number of patients** | **Age range** | **Study centre** | **Imaging (x-rays/CT) Scanner** | **Examination parameters** | **Chest severity score calculation** | **Main results** | **Study design** |
| --- | --- | --- | --- | --- | --- | --- | --- | --- |
| **Al-Mosawe et al., 2021** (1) | 100 males, and 73 females (total = 173) | 20-85 years | Single centre (Baghdad) | Chest CT scan using Philips GEMINI TF **64 slice** | Axial with 3 mm slice thickness, FOV= 400 mm, Tube current = 20–30 mA, tube voltage = 120–140 kV, matrix = 512, pitch = 1.078, and rotation time = 0.75 s | An expert radiologist analysed and assessed each CT scan.  The severity score was determined based on the percentage of lung involvement in each patient, with the percentage of each lobe involved being scored. | Ground glass opacities were the most frequently observed pattern of lung alterations, occurring in 79% of cases. There were significant positive relationships between CTSS levels and male gender and age. Significant link was found between the amount of lung involvement on a CT scan and positive PCR test findings (p = 0.001917); as the CT severity index increases, the likelihood of a positive PCR test increases. | Prospective cross-sectional study |
| **Han et al., 2021** (2) | 114 COVID patients (F=30%, M=70%) | 24-82 (mean=54) | Single study  (Wuhan Jin Yin-tan Hospital) | Both the SOMATOM Definition AS+ and SOMATOM Perspective CT scanners were used to perform the scans (Siemens Healthineers, Forchheim, Germany). | Initial CT exams were conducted with the patient in the supine position. Chest CT scans without contrast were acquired from the thoracic inlet to the diaphragm. The following characteristics were utilized: 640.6 mm or 1280.6 mm detector collimation widths and a tube voltage of 120 kV. The tube current was controlled by an exposure control system. Images of 62/114 (54%) patients were rebuilt with a 5mm slice thickness and a 5mm spacing. 52/114 (46%) individuals had their images reconstructed with a slice thickness and interval of 1mm. | To quantify the extent of pulmonary abnormalities (total lesions, GGO, consolidation, reticulation, and fibrotic-like changes), a semiquantitative CT score was assigned on the basis of the area involved in each of the five lung lobes: 0= no involvement; 1= < 5% 2= 5%-25%; 3= 26%-49%; 4= 50%-75%; and 5= >75%. The total CT severity score was calculated by summing the individual lobar scores (possible scores range from 0 to 25). | On follow-up CT, evidence of fibrotic-like changes was observed in 40/114 (35%) of patients (group#1), while the remaining 74/114 (65%) patients (group#2) showed either complete radiological resolution (43/114, 38%) or residual ground-glass opacification or interstitial thickening (31/114, 27%). Multivariable analysis identified age >50 years (odds ratio [OR]:8.5, 95%CI:1.9-38, p=.01), heart rate >100bpm at admission (OR:5.6, 95%CI:1.1-29, p=.04), duration of in-hospital stay ≥17 days (OR:5.5, 95%CI:1.5-21, p=.01), and acute respiratory distress syndrome (OR:13, 95%CI:3.3-55, p<.001), non-invasive mechanical ventilation (OR:6.3, 95%CI:1.3-30, p=.02) and total CT score ≥18 (OR:4.2, 95%CI:1.2-14, p=.02) on initial CT as independent predictors for lung fibrotic-like changes at 6 months. | Prospective longitudinal design |
| **Omar et al., 2020** (3) | 19 males, and 11 females (total =30) | 26-63 years  (Mean age= 43) | Single centre in Egypt | High resolution CT  - no details of CT slice are given | -All images were obtained with the patients in the supine position. Scans were done from the level of the upper thoracic inlet to the inferior level of the costophrenic angle  -Tube voltage 120 kVp, mAs 250, detector collimation width 2 × 0.25, rotation time 1.0 s, and slice thickness 2.5 mm. | - All CT images were analysed by 3 radiologists with working experience from 15-20 years. Each of the five lung lobes was assessed for the degree of involvement and classified as none (0%), minimal (1–25%), mild (26–50%), moderate (51–75%), or severe (76–100%). | The most common CT features detected in COVID-19 cases were ground glass patches (93.3%), subpleural linear abnormality (53.3%), air bronchogram (23.3%), and consolidation patches and bronchial wall thickening (16.7%), crazy paving pattern (13.3%), and discrete nodules surrounded by ground glass appearance (10%). Only one case had pleural effusion (3.3%). most patients (>66%) had 6-10 scores out of 20 (total severity score) and most of them (>30%) had 3 affected lung lobes. | Retrospective study |
| **K. Li et al., 2020** (4) | 38 males, and 40 females (total = 78) | Mean= 44.6 years | Single centre on two CT scanners in Zhuhai, China. | Two CT scanners, an uCT 760 and an uMI 780, were used for chest imaging. | Without intravenous contrast, all scans were conducted with the patient in the supine position during end-inspiration. The scanning range extended from the lung's apex to its base. All pictures were acquired with a slice thickness of 1.0 mm, an increment of 1.0 mm, a resolution of 512 mm 512 mm, and a crisp reconstruction kernel. With a window level of 600 Hounsfield units (HU) and a window width of 1500 HU, the lung window was set. | 3 radiologists analysed and reviewed all CT images.  - Each of the five lung lobes was assessed for the degree of involvement and classified as none 0 (0%), minimal 1 (1–25%), mild 2 (26–50%), moderate 3 (51–75%), or severe 4 (76–100%). | The median TSS of severe-critical-type group was significantly higher than common type (pts with +CT findings and different clinical manifestations). CT visual quantitative evaluation has high consistency (ICC >.976) among the observers. ROC analysis showed the AUC of TSS for diagnosing severe-critical type was 0.918. The TSS cut-off of 7.5 had 82.6% sensitivity and 100% specificity. The proportion of confirmed COVID-19 patients with normal chest CT was relatively high (30.8%); CT was not a suitable screening modality. | Retrospective study |
| **Yang et al., 2020** (5) | 102 (49 females, 53 males) | 15-79 years | Gorges hospital (China) | CT using a 16-detector CT scanner (Emotion; Siemens). | All patients were examined in the supine position. CT images were then acquired during a single inspiratory breath-hold. The scanning range was from the apex of the lung to the costophrenic angle. CT scan parameters were as follows: x-ray tube parameters 120 kVp, 350 mAs; rotation time 0.5 second; pitch 1.0; section thickness 5 mm; intersection space 5 mm; additional reconstruction with a sharp convolution kernel; and a slice thickness of 1.5 mm. | 2 radiologists with more than 10 years of experience reviewed CT images.  Using the anatomical structure, this study may divide the 18 lung segments into 20 regions: apical and posterior for the posterior apical segment of the left upper lobe, and anterior and basal for the anteromedial basal segment of the left lower lobe. On chest CT scans, opacities in each of the 20 lung regions were subjectively scored as 0, 1, or 2 depending on whether or not the opacification involved parenchymal tissue (i.e., the lung tissue). To calculate the CT-SS, researchers added up the possible values (from 0 to 40) in each of the 20 lung segment regions. | The posterior segment of upper lobe (left, 68 of 102; right, 68 of 102), superior segment of lower lobe (left, 79 of 102; right, 79 of 102), lateral basal segment (left, 79 of 102; right, 70 of 102), and posterior basal segment of lower lobe (left, 81 of 102; right, 83 of 102) were the most frequently involved sites in Covid-19. Lung opacification mainly involved the lower lobes, in comparison with middle-upper lobes. No significant differences in distribution of the disease were seen between right and left lungs. The individual scores in each lung and the total CT-SS were higher in severe COVID-19 when compared with mild cases (*P* < .05). The optimal CT-SS threshold for identifying severe COVID-19 was 19.5 (area under curve = 0.892), with 83.3% sensitivity and 94% specificity. | Retrospective study |
| **Saeed et al., 2021** (6) | 902 patients (769males, and 133 females | 19-87 years  (Mean age= 44.2) | single centre (Sheikh Khalifa Medical City, Abu Dhabi UAE) | VCT GE **64 slice** scanner | -All images were obtained with the patients in the supine position with single breath hold. parameters were as follows: scan direction (craniocaudally), tube voltage (120 kV), tube current (100–600 mA)-smart mA dose modulation, slice collimation (64 × 0.625 mm), width (0.625 × 0.625 mm), pitch [[1](https://www.ncbi.nlm.nih.gov/pmc/articles/PMC7801942/#B1)], rotation time (0.5 s), and scan length (60.00–I300.00 s). | 2 radiologists with 8 years of working experience determined the disease severity score in each patient. Severity was assessed using the scoring system 1- 5 which depends on the visual assessment of each lobe involved. | CT severity score was found to be positively correlated with lymphopenia, increased serum CRP, d-dimer, and ferritin levels (p < 0.0001). The oxygen requirements and length of hospital stay were increasing with the increase in scan severity. | Retrospective study |
| **Borghesi et al., 2020** (7) | 532 males, and 251 females (total = 783) | 20-89 years Mean= 44.6 years | Single X-ray centre | Chest x-ray reports containing the new scoring system | CXR reports containing the new scoring system | 1 radiologist analysed and reviewed all CXR reports.  - Each of the five lung lobes was assessed for the degree of involvement and classified as none 0, minimal 1, moderate 2, severe 3. | The CXR score was significantly higher in males than in females only in groups aged 50 to 79 years. A significant correlation was observed between the CXR score and age in both males and females. Males aged 50 years or older and females aged 80 years or older with coronavirus disease 2019 showed the highest CXR score or highest risk of developing severe lung disease. | Retrospective study |
| **Borghesi & Maroldi, 2020** (8) | 902 patients (769males, and 133 females) | 19-87 years  (Mean age= 44.2) | single centre (Sheikh Khalifa Medical City, Abu Dhabi UAE) | VCT GE **64 slice** scanner | -All photos were taken with the patients lying supine and holding a single breath. Scan direction (craniocaudally), tube voltage (120 kV), tube current (100-600 mA)-smart mA dose modulation, slice collimation (64 0.625 mm), width (0.625 0.625 mm), pitch rotation time (0.5 s), and scan length (60.00-I300.00 s) were the parameters. | 2 radiologists with 8 years working experience determined the disease severity score in each patient. Severity was assessed using the scoring system 1- 5 which depends on the visual assessment of each lobe involved. | CT severity score was found to be positively correlated with lymphopenia, increased serum CRP, d-dimer, and ferritin levels (p < 0.0001). The oxygen requirements and length of hospital stay were increasing with the increase in scan severity. | Retrospective study |
| **Lieveld et al., 2021** (9) | 741 (417 M) | 62.1 years |  |  |  |  | The results showed that utilizing PCR as a standard, the AUC for CO-RADS was 0.91 (95% CI, 0.89-0.94). With a sensitivity of 89.4 percent (confidence interval [CI], 84.7-93.0) and a specificity of 87.2 percent, the best CO-RADS cut-off was 4. (CI, 83.9-89.9). Adjusted odds ratios (ORs) per point increase in CTSS for hospitalization, intensive care unit (ICU) admission, and 30-day mortality were 1.19 (95% CI: 1.09-1.28), 1.23 (1.15-1.32), and 1.14 (1.07-1.22), respectively. The inter-quartile ranges for the intraclass correlation coefficients of the CO-RADS and CTSS were 0.94 (0.91-0.96) and 0.82. (0.70-0.90). Based on these results, CO-RADS and CTSS should be used to triage, diagnose, and manage patients presenting to the ED with suspected cases of COVID-19. | Prospective |
| **Francone et al., 2020** (10) | 325 symptomatic patients (64.6% M, 35.5% F) | 25-75 | Italy (emergency hospital?) | All examinations were performed using two multidetector CT scanners (Somatom Sensation 16 and Somatom Sensation 64; Siemens Healthineers). | Scanning parameters were identical to the manufacturer’s standard recommended pre-setting for a thorax routine. Images were reconstructed with a 1-mm slice thickness in all cases using the classic filtered back-projection method with a soft tissue kernel of B20 and a lung kernel of B60. Coronal and sagittal multiplanar reconstructions were also available in all cases. | Based on the degree of anatomical involvement, a semi-quantitative CT severity score was determined for each of the five lobes in all instances, as proposed by Pan et al. Engagement levels range from 0% (no involvement) to 5 (very significant involvement) with 1 being no involvement and 5 being involvement of over 75%. After adding up the scores from each lobar region, the final global CT score was (0 to 25). | Early-phase disease (less than 7 days since symptoms began) was characterized by ground glass opacities, while late-phase disease (more than 7 days since symptoms began) was marked by a crazy-paving pattern, consolidation, and fibrosis. Patients in the late stages had higher CT scores than those in the early stages, and patients in the critical and severe stages had higher CT scores than those in the mild stage (p 0.0001). CT score was substantially linked with CRP (p < 0.0001, r = 0.6204) and D-dimer (p < 0.0001, r = 0.6625) levels. Multivariate analysis (HR, 3.74; 95% CI, 1.10-12.77; p = 0.0348) and univariate analysis (HR, 8.33; 95% CI, 3.19-21.73; p 0.0001) both indicated that a CT score of 18 was predictive of death. | Retrospective |
|  | 926 consecutive patients | Median age= 69 years | Two-centre study in Northern Italy | X-rays machine | Chest x-ray was performed.  Three x-ray machines were used. At centre 1, two separate CXR systems were used (Digital GM85, Samsung Healthcare, Seoul, South Korea; Digital FDR Go PLUS, Fujifilm, Tokyo, Japan) while at centre 2, just one system was used to perform CXRs at the bedside in the ED isolation rooms (Easyslide 30, SMAM, Monza, Italy). | All anonymized and randomized CXRs from the two centres were independently and blindly reviewed by five readers: two radiologists from centre 1 and three radiologists from centre 2 with 6-15 years of experience in chest imaging. To provide a semiquantitative severity score, readers divided each lung into three zones (1): upper (from the lung apex to the aortic arch profile), (2) middle (from the aortic arch profile to the lower edge of the left pulmonary hilum), and (3) lower (from the lower margin of the left pulmonary hilum to the diaphragm). Each zone was given a score between 0 and 3 with increments of 1. 0: Normal lung parenchyma; 1: Interstitial Involvement Only; 2: Radiopacity Present in Less Than Half of Visible Lung Parenchyma; 3: Radiopacity Present in Half or More of Visible Lung Parenchyma. | The median severity score was significantly higher in patients who ultimately passed away (p = 0.003), and it correlated negatively with oxygen saturation (= -0.242, p 0.001), positively with white blood cell count ( = 0.277, p 0.001), lactate dehydrogenase ( = 0.308, p 0.001), and C-reactive protein ( = 0.367, p 0.001). Interobserver agreement ranged from moderate ( = 0.449, p 0.001) to almost perfect ( = 0.872, p 0.001) when total scores were considered; readers from centre 2 agreed more strongly (up to = 0.872, p 0.001) than readers from centre 1 ( = 0.764, p 0.001). | Retrospective study |
| Steinbeis et al. 2022 (11) | 180 (68 females and 112 males) COVID-19 patients | Mean= 56.50 years | Single study at Charité Universitätsmedizin Berlin, an academic tertiary care medical centre | CT scan | A routine chest CT scan was performed. | Two senior thoracic radiologists reviewed chest-CT scans. All CXRs were blinded to patient clinical features and disease severity. A visual score ranging from 0 (no involvement) to 5 (>75% involvement) to measure acute pulmonary involvement was used. | Patients' median age was 57; 37.8% were female. Age, male sex, and BMI were linked with acute-COVID-19 severity (p <0.05). Disease severity was also linked to pulmonary limitation and CO2 diffusion. In patients with limitation and decreased diffusion capacity, FVC improved from 61.32 to 71.82, TLC from 68.92 to 76.95, DLCO from 60.18 to 68.98, and KCO from 81.28 to 87.80 (p<0.05). CT-score of acute lung involvement was associated with reduced diffusion capacity. High-severity patients' respiratory symptoms improved throughout follow-up, but not mild patients. | Prospective longitudinal study |
| Wong et al. 2020 (12) | 64 COVID-19 patients (26 men) | mean age 56±19 years | - Multiple centre  - four tertiary and regional hospitals in Hong Kong (Queen Mary Hospital, Pamela Youde Nethersole Eastern Hospital, Queen Elizabeth Hospital, and Ruttonjee Hospital) | X-ray machine | Chest x-ray | Each lung was given a score between 0 and 4 based on the percentage of consolidation or GGO present: 0 for no consolidation or GGO, 1 for 25% to 75%, 2 for 50% to 75%, and 4 for >75%. To determine the overall severity, we added together the results for both lungs. | Positive first RT-PCR was found in 58 of these patients (91%, [CI: 81-96%]), abnormal baseline CXR was found in 69 of these patients ([CI: 56-80%]), and 59 of these patients had both a positive initial RT-PCR and an abnormal baseline CXR ([CI:46-71%]). CXR abnormalities were present in 6/90 patients (9%) before RT-PCR was positive. Compared to the baseline CXR (69% [95% CI: 56-80%]), the sensitivity of the initial RT-PCR was higher (91% [95% CI: 83-97%]; p = 0.009). Recovery on imaging studies (mean 6 5 days) and virus testing (mean 8 6 days) did not differ statistically significantly (p= 0.33). The most frequent discovery was consolidation (47%; 30/64), followed by GGO (33%; 21/64). Peripheral (26/64, 41%) and lower-zone (32/64, 50%) abnormalities on CXR were most common, and the majority of cases were bilateral (32/64, 50%). Just 2% of people with 64 checked had pleural effusion. Ten to twelve days after the onset of symptoms was when the CXR readings were at their worst. | Retrospective study |
| Mruk et al. 2021 (13) | 77 consecutive patients |  | - single centre at the Central Clinical Hospital of the Ministry of the Interior in Warsaw | CT scan | Patients were scanned on a 16-detector CT scanner while lying supine for a single inspiratory breath-hold, with the scanning range beginning at the apex of the lungs at the costophrenic angle. The CT scan was performed at 80 to 120 KVp, 100 mAs, 1.2 mm slice thickness, and 1.2 mm pitch. A workstation running OsiriX MD v.8.0.2 software was used to examine the CT scans for lung parenchyma, with a window width of 1500 HU and a level of 600 HU. | Two radiologists assessed the scans independently. Chest CT scans were evaluated using three different scales: the Total Severity Score, which assigned a value between 0 and 20 points to each of the five lung lobes (for a total score of 0 to 20 points), the Chest CT Score, which assigned a value between 0 and 25 points to each of the five lung lobes, and the Chest CT Severity Score, which assigned a value between 0 and 2 points to each of the 20 lung segments (total score of 0 to 40 points). | It was shown that (1) the κ was 0.944 and the ICC was 0.948 for the total severity score; (2) the κ was 0.909 and (3) the ICC was 0.919 for the CT-severity; and the κ was 0.888 and the ICC was 0.899 for the CT-severity score. In addition, the CT-SS had the highest positive connection with the patient's clinical status as reflected by the Modified Early Warning Score (r=0.627 for Radiologist 1 and r=0.653 for Radiologist 2). | Retrospective study |

References

1. Al-Mosawe AM, Abdulwahid H mohammed, Fayadh NAH. Spectrum of CT appearance and CT severity index of COVID-19 pulmonary infection in correlation with age, sex, and PCR test: an Iraqi experience. The Egyptian Journal of Radiology and Nuclear Medicine. 2021;52(1):40.

2. Han X, Fan Y, Alwalid O, Li N, Jia X, Yuan M, et al. Six-Month Follow-up Chest CT findings after Severe COVID-19 Pneumonia. Radiology. 2021 Jan 26;203153.

3. Omar S, Motawea AM, Yasin R. High-resolution CT features of COVID-19 pneumonia in confirmed cases. Egyptian Journal of Radiology and Nuclear Medicine. 2020 Jul 7;51(1):121.

4. Li Y, Xia L. Coronavirus Disease 2019 (COVID-19): Role of Chest CT in Diagnosis and Management. AJR Am J Roentgenol. 2020 Jun;214(6):1280–6.

5. Yang R, Li X, Liu H, Zhen Y, Zhang X, Xiong Q, et al. Chest CT Severity Score: An Imaging Tool for Assessing Severe COVID-19. Radiology: Cardiothoracic Imaging. 2020 Apr;2(2):e200047.

6. Saeed GA, Gaba W, Shah A, Al Helali AA, Raidullah E, Al Ali AB, et al. Correlation between Chest CT Severity Scores and the Clinical Parameters of Adult Patients with COVID-19 Pneumonia. Radiology Research and Practice. 2021 Jan 8;2021:e6697677.

7. Borghesi A, Zigliani A, Masciullo R, Golemi S, Maculotti P, Farina D, et al. Radiographic severity index in COVID-19 pneumonia: relationship to age and sex in 783 Italian patients. Radiol Med. 2020 May;125(5):461–4.

8. Borghesi A, Maroldi R. COVID-19 outbreak in Italy: experimental chest X-ray scoring system for quantifying and monitoring disease progression. Radiol Med. 2020 May;125(5):509–13.

9. Lieveld AWE, Azijli K, Teunissen BP, van Haaften RM, Kootte RS, van den Berk IAH, et al. Chest CT in COVID-19 at the ED: Validation of the COVID-19 Reporting and Data System (CO-RADS) and CT Severity Score. Chest. 2021 Mar;159(3):1126–35.

10. Francone M, Iafrate F, Masci GM, Coco S, Cilia F, Manganaro L, et al. Chest CT score in COVID-19 patients: correlation with disease severity and short-term prognosis. Eur Radiol. 2020 Dec;30(12):6808–17.

11. Steinbeis F, Thibeault C, Doellinger F, Ring RM, Mittermaier M, Ruwwe-Glösenkamp C, et al. Severity of respiratory failure and computed chest tomography in acute COVID-19 correlates with pulmonary function and respiratory symptoms after infection with SARS-CoV-2: An observational longitudinal study over 12 months. Respir Med. 2022 Jan;191:106709.

12. Wong HYF, Lam HYS, Fong AHT, Leung ST, Chin TWY, Lo CSY, et al. Frequency and Distribution of Chest Radiographic Findings in COVID-19 Positive Patients. Radiology. 2020 Mar 27;201160.

13. Mruk B, Plucińska D, Walecki J, Półtorak-Szymczak G, Sklinda K. Chest Computed Tomography (CT) Severity Scales in COVID-19 Disease: A Validation Study. Med Sci Monit. 2021 May 5;27:e931283-1-e931283-6.
